# Ethical challenges experienced by UK military medical personnel deployed to Sierra Leone (operation GRITROCK) during the 2014–2015 Ebola outbreak: a qualitative study

**DOI:** 10.1186/s12910-017-0234-5

**Published:** 2017-12-19

**Authors:** Heather Draper, Simon Jenkins

**Affiliations:** Health Sciences, Warwick Medical School, University of Warwick, Gibbet Hill, Coventry, CV4 7AL UK

**Keywords:** Ebola virus disease, Military medical ethics, Ethics, Disaster ethics, Infectious disease outbreaks, Military humanitarian interventions, Qualitative research, Empirical ethics, Medical rules of eligibility

## Abstract

**Background:**

As part of its response to the 2014 Ebola outbreak in west Africa, the United Kingdom (UK) government established an Ebola treatment unit in Sierra Leone, staffed by military personnel. Little is known about the ethical challenges experienced by military medical staff on humanitarian deployment. We designed a qualitative study to explore this further with those who worked in the treatment unit.

**Method:**

Semi-structured, face-to-face and telephone interviews were conducted with 20 UK military personnel deployed between October 2014 and April 2015 in one of three roles in the Ebola treatment unit: clinician; nursing and nursing assistant; and other medical support work, including infection control and laboratory and mortuary services.

**Results:**

Many participants reported feeling ethically motivated to volunteer for deployment, but for some personal interests were also a consideration. A small minority had negative feelings towards the deployment, others felt that this deployment like any other was part of military service. Almost all had initial concerns about personal safety but were reassured by their pre-deployment 'drills and skills', and personal protective equipment. Risk perceptions were related to perceptions about military service. Efforts to minimise infection risk were perceived to have made good patient care more difficult. Significantly, some thought the humanitarian nature of the mission justified tolerating greater risks to staff. Trust in the military institution and colleagues was expressed; many participants referred to the ethical obligation within the chain of command to protect those under their command. Participants expected resources to be overwhelmed and ‘empty beds’ presented a significant and pervasive ethical challenge. Most thought more patients could and should have been treated. Points of reference for participants’ ethical values were: previous deployment experience; previous UK/National Health Service experience; professional ethics; and, distinctly military values (that might not be shared with non-military workers).

**Conclusion:**

We report the first systematic exploration of the ethical challenges face by a Western medical military in the international response to the first major Ebola outbreak. We offer unique insights into the military healthcare workers’ experiences of humanitarian deployment. Many participants expressed motivations that gave them common purpose with civilian volunteers.

## Background

In July 2014, the World Health Organisation (WHO) acknowledged the seriousness of the Ebola virus disease (EVD) outbreak in West Africa, and called on governments around the world to respond with humanitarian aid and to take action to contain the spread [1]. The United Kingdom (UK) government, through the Department for International Development (DFID), and working with Save the Children, deployed its military (Operation GRITROCK) to Sierra Leone in a variety of roles, one of which was to staff a small, well equipped Ebola treatment unit for international and local healthcare workers (and other EVD-infected foreign nationals), [2] co-located with a larger Ebola unit for the general population that would be staffed by a non-governmental organisation (NGO). Two parallel military medical infrastructures were deployed: one to provide general health services for military personnel (and other eligible persons) and the other specifically and only to treat EVD cases in the treatment unit. According to Bricknell et al., [3] the goal for the Ebola treatment unit was “to demonstrably deliver a level of care to infected healthcare workers and other entitled patients as close as safely practicable to that provided in Western national infectious disease containment facilities.” The Medical Rules of Eligibility (MRoE),^1^ “covered the international community deployed in support of the wider Ebola crisis, which included those facilities contracted by DFID and other international healthcare workers employed in ETCs [Ebola Treatment Centres]” and also gave the most senior Commander Medical “the discretion to accept admissions for Sierra Leonean healthcare workers” capacity permitting [3].

The original plan was for a single deployment to the treatment unit lasting 60 days. In the event, several different tranches were deployed between October 2014 and July 2015 [3].

Very little literature exists exploring the ethical challenges experienced by humanitarian workers [4–6] and even less is known about the experiences of the medical military employed in an humanitarian capacity, though there is an established body of literature on military medical ethics in combat and peacekeeping scenarios [7, 8]. Draper (HD), a civilian Professor of Bioethics, had been collaborating in various ways over for several years with the Royal Centre for Defence Medicine (Academia and Research) (RCDM) to improve understanding of, and training in, military medical ethics. After the withdrawal of British troops from Afghanistan, there were plans in place to design ethics training for potential humanitarian deployment as the UK military moved into contingency following the withdrawal. When the UK Ebola response was announced, it was recognised that this would be a unique opportunity to gain an understanding of the ethical challenges faced by a humanitarian military deployment. Accordingly, a joint application for funding was made to the Economic and Social Research Council and unprecedented access to participants for this study was facilitated by RCDM. It was also recognised that whilst the Ebola outbreak was an extreme scenario it had elements in common with other mass infectious disease outbreaks and other events (e.g. chemical or radiological contamination) where responding posed significant risks to medical personnel.

## Methods

The key objectives for this qualitative study were to identify and explore the ethical challenges the military personnel working in the Ebola treatment unit felt they had faced, and to understand how they responded to these with a view to improving preparation and training for future humanitarian deployments.

Participants were recruited by email between March and July 2015. Sampling was purposive to cover role-group and timing of deployment. Potential participants were identified (by a RCDM post-doctoral military nurse researcher with expertise in qualitative methods) by deployed role within three broad groups: clinical/doctor; nurse/nursing assistant; and medical support, including laboratory, infection control, personal protective equipment (PPE) monitors, and mortuary attendants etc. This enabled us to capture a wide range of experiences across medical and military hierarchies. All personnel deployed to the treatment unit, and having returned from deployment during the recruitment period, were included. The email, sent by the same military member of staff, contained an invitation letter from HD (the principal investigator), the participant information sheet and sample consent form. Recipients were asked to respond directly to HD if they were interested in participating. All participants were offered a face-to-face interview but some preferred to be interviewed by telephone. Consent was obtained and recorded immediately prior to the interview. No military record of those participating was kept (nor were the identities of any of those participating shared with military colleagues) and a reminder was therefore sent to all after two weeks, by the same military member of staff. The voluntary nature of participation was stressed and the rank of the military originator of the email was removed to avoid any perception of coercion.

20 semi-structured interviews were conducted in the UK March – August 2015 by a single investigator (HD), using a topic guide with open-ended questions from which the interviewee and interviewer were free to depart. The interviews were structured to cover three areas of experience: perceptions from the point of receiving deployment orders to the end of pre-deployment training; perceptions whilst on deployment, including the identification of common or especially troubling ethical challenges; and, finally participants’ reflections on their experience subsequent to their return. The interviews were audio-recorded and transcribed verbatim.

The interviews were coded independently by Jenkins (SJ) and HD. When both had completed coding nine interviews, they met to discuss the emerging codes and to compare coding on a single transcript, as a way of bolstering intercoder reliability [9]. A preliminary coding scheme was then developed. The coded transcriptions were checked against this and then the outstanding transcriptions were coded. A further meeting was held to discuss and agree the final coding scheme. This resulted in single data set, managed using NVivo software ‘to facilitate an accurate and transparent data analysis process’, [10] with adjustments being made to the coding following discussion. After further discussion, the codes were grouped into categories that best reflected the patterns emerging from the data, and key overarching concepts were selected. These were then discussed with the project advisory group. The advisory group comprised one independent, senior academic specialist in disaster bioethics and also knowledgeable about the research methods used (Professor Lisa Schwartz, McMaster University, Canada), the then Medical Director of RCDM (Brigadier Timothy Hodgetts) and an experienced Deployed Medical Director (Colonel Jeremy Hemmings) who chaired the meetings.

This thematic analysis mapped many of the stages of the process described by Braun and Clarke [11]. Given the dearth of literature on these ethical issues, we took a largely conventional approach to content analysis [11] using the data to draw conclusions about themes in the participants’ thinking but not trying to construct a unified overarching theory to explain these as per a grounded theory approach [12]. The results were presented to selective participants for member validation.

## Results

The numbers of participants, reported by role-group and period of deployment, and interview length, are shown in Table 1. Face to face interviews tended to be longer than telephone interviews, though the shortest interview was one conducted face-to-face.

**Table 1:** Participant characteristics and interview length

Deployed role	Doctors	Nursing	Other		
Total 20	7	6	7		
Deployed	Oct-Dec 2014	Dec- Mar 2015	April–July 2015	n/a	
	7	11	1	1	
Interviews	Shortest	Longest	Mean	45–75 min	Face-to-face
	31 mins	208 mins	80 mins	10	11

From this rich data set, this paper reports on the broader ethical challenges that the participants experienced. Our findings are illustrated with selected quotations that reflect typical responses and the range of participants within each of the three broad groups. To protect participants’ identity, given the relatively small population size, we have not included rank or role using instead a simple numbering system (P1, P2 etc.). To avoid repetition, asterisks (^* ** ***^ etc.) are used to identify particular quotations and participants that are referred to again later.

### Attitudes to and motivations for deploying

Many participants regarded themselves as having volunteered for deployment. Indeed, some participants reported making strenuous efforts to go. This may have been a misperception given that, as one participant explained: "*It’s not done as ‘Are you willing to go because if you are not you don’t have to’ ...[it’s] only used in order to prioritise people...so the idea of volunteering...it’s a bit of a misnomer."* (P17) Nonetheless, for these participants, the decision to volunteer was an ethical one. The majority felt compelled to respond to the unfolding humanitarian crisis and human suffering it was generating. Some cited having the skills/ability to make a difference as a motivation and one participant reported feeling a personal affiliation with the African people.

*...it was very clear that there was a, a desperate humanitarian crisis going on and so there was a desperate need.* (P12).

*I was watching this suffering on the TV… I’d watched the TV and think, ‘I'm a nurse, an experienced nurse,’ and I, I, I sort of knew in my heart. I ... really wanted to do something. I knew I had the skills, as it were, to alleviate suffering.* (P7).

A significant minority sought to deploy primarily as a career opportunity, sometimes related to their medical speciality or command/rank ambitions, or they simply welcomed the opportunity to deploy.

*I was immediately interested ... but only in a role that is relevant to my rank, because what I didn’t want to be doing was taking time out from work or from my reservist role if I felt that it was ... a role that wasn’t also going to career develop me and professionally develop me... I was desperately keen to go but I wanted to make it the best opportunity possible ... and which will then translate to skills that I bring back into my workplace.* (P12*).*

Rarely did participants provide a single motivation and most cited a combination of the above reasons (as P12 above). Of those who did not report actively seeking to deploy, most reported feeling sanguine about the prospect, regarding deployment as part of military life; others were part of a high readiness unit and were therefore already on notice to deploy. A small minority reported being very negative about the prospect, feeling that this type of mission was not what they signed up for when they joined the military.

*I wasn’t given the order to go, but I am in the military and that’s what’s expected of me.* (P11).

*...as a military or army medic we all joined up to maintain the fighting strength of the British Army and this just wasn’t anything to do with that.* (P5*).

This view is in contrast to those who reported joining the military to do humanitarian work and/or being disappointed by how few opportunities had arisen to do so.

*I thought I was joining up to travel round the world and to save people...I’ve done nearly eighteen years now and this is the first humanitarian work I’ve done...when I was joining up I thought we would be doing a lot more disaster relief kind of work.* (P19).

Only a very few participants made no reference to concerns about the personal risks involved, most expressed at least initial concerns. All of the participants expressed confidence in the PPE training they had received and found this reassuring. Some referred explicitly to having trust in the military to look after them, and one reservist reported that whilst they had volunteered to deploy with the military they would not have gone as a National Health Service (NHS) volunteer.

*Sheer terror because I thought it was operation certain death.* (P5).

*...it would always be one of those things that would happen – which would be human error rather than actually anything you hadn’t done yourself to prevent...we were taught very much to be very sensible...not to take any risks and to practice our drills...But there are obviously occasions that are completely out of your hands, when dealing with patients that are confused or agitated...* (P21).

*I wasn’t really worried about catching Ebola because I did have a lot of trust in training we had and I felt confident that as long as I stuck to my ‘drills and skills’ and we looked after each other, we’d be fine.* (P13***).

### Perceptions of ‘the mission’ and concerns for personal safety

The participants provided a uniform account of the mission, namely that they were deploying to provide high quality care to EVD-infected healthcare workers so as to bolster confidence internationally (other countries would send medical teams to help) and locally (healthcare professionals would continue working knowing they would get treated if they became infected); some also made reference to containing the spread of EVD.

*Well all the civilian healthcare people out there, they are basically like ‘If the army aren’t there, then we’re not going to be here’. So if the army aren’t there then the healthcare professionals won’t be there, so then no one will get treated...and then it’s just going to be...a massive pandemic of Ebola...it’s just going to go global.* (P16).

Their perception of the mission, however, varied. Some participants believed that they were embarked on a fundamentally humanitarian mission whilst others regarded it as a non-combat contingency mission. Participants often suggested that that there was an overriding desire from the top of the chain of command to minimise the risk that personnel would be infected.

*Whereas the army certainly has not done a humanitarian mission like this before I don’t think and has not done much in the way of humanitarian, medical humanitarian stuff for a long time*. (P1).

*...it was more interesting because it was something so different and we hadn’t done a contingency op before so that made it a bit more interesting but generally not more important I don’t think than previous deployments.* (P15).

*...people senior to us were terrified of military personnel contracting Ebola or dying out in Africa or over-stretching resources back in the UK.* (P5).

Participants’ understanding of the nature of the mission, however, affected their views on how minimal risk should be conceived. Successfully delivering the mission and minimising risk was experienced as a significant ethical challenge. The participants’ perceptions about risk helped to inform our understanding of why this was, as we shall now explain.

No participants seemed inherently risk-adverse, and several stated explicitly that risk-taking is part and parcel of what the military do.

*...if you join the Army you are expecting to get sent into risky places and the, the whole purpose of the Army is so that we can take that risk and so that the UK can remain safe. I mean that’s the whole point about having an army at all is, is for the promotion of the safety of home. So, I personally don’t think that anyone who was deploying should have felt that their safety was above that of... the population back at home.* (P17**).

Equally, a minority felt that they had not ‘signed up for’ these particular risks, suggesting that risk-perception was related closely to their understanding of the justification for risk-taking (see P5* above). Some thought that the risk from EVD was a *different* sort of risk to that normally taken without necessarily suggesting that it was therefore a *greater* risk. Reference was made to infection being a risk that could not be seen, unlike normal combat risks.

*Ebola is an unseen killer. You can’t see it and of course what we’re used to in a trauma-type environment is, is things which are very visible. So the fact that it is...it is invisible it’s scary. You know there’s no, there’s no doubt about it, it’s scary.* (P12).

This observation was probed in later interviews because it was not obvious that normal military risks are clearly visible; snipers, landmines and improvised explosive devices (IEDs), for instance, are most effective when their position has not been detected. Moreover, larger medical units are generally located at a relatively safe distance from combat operations. Accordingly, it seemed to us that combat duties may also entail an element of ever-present but unseen danger. Probing brought some clarity to the concerns being expressed. The sense that the risk was invisible was heightened because one might be infected for several days before realising it. Bullets and explosives leave obvious injuries that can then be responded to; the gravity or otherwise of these is more immediately clear, and the treatment pathways more familiar.

*one of the guys said, that he thought it was much worse than being on patrol; not because it was actually more dangerous but that if he was on patrol and he stepped on a landmine, he knew about it, whereas, if he caught Ebola, he wouldn’t know about it till the week later, which was – which a lot of us found a very disturbing concept, once we started thinking about that... it’s almost better to be harmed in a way that you’re aware of immediately than to have something in your body that’s harming you and you don’t know, you’re either harmed or you’re not harmed ... when you’re on the ground. ... we had exposures to Ebola, there were a lot of people who found that very difficult; the idea of, ‘I might actually have caught this yesterday and I just don’t know it yet’ ...a lot of people said they found that very uncomfortable; the not knowing if they’d already made the mistake.* (P18).

Some participants who regarded the mission as primarily a humanitarian one felt the scale of the outbreak justified a higher risk threshold. Other participants also felt that the risk-aversion governing the mission ran counter to the general willingness to expose military personnel to risk in combat operations.

*Our mission wasn’t to go there and not get infected, our mission was to go there and have a safe ETU* [Ebola treatment unit] *to treat healthcare workers and we’ve done that and if the mission is important enough to have to acknowledge some risk there and people think, if they think you can eliminate risk in looking after people with Ebola down there, it’s impossible and you can’t and you will always have a human factor or a human error as how someone will get infected and that’s a disaster but if you believe in the overall mission, which I do, then I think it’s justified.* (P2).

*We are always quoted a figure of risk when we deploy. But you know people have deployed for the last ten years, into areas where they are risking their lives, and we are medics, we are doctors. What’s the difference? We are all in the same organisation and it’s a risk.* (P21).

Some staff with specialist knowledge in infectious disease or managing contamination thought the risks of becoming infected whilst wearing PPE were over-stated. As we have already noted, generally staff felt confident that they were fairly safe if they followed their training.

*I must admit I wasn’t as nervous about this deployment because I think I’d managed to put the disease into perspective before I’d even got there and sort of said statistically it’s actually quite low risk so long as you are sensible in those key moments when you might be dealing with a patient*. (P10).

Several mentioned the ‘body-mapping’^2^ exercise [13] during training and had found this very reassuring. Participants who deployed during the period when, in rapid succession, one colleague was confirmed to be EVD positive and two others experienced needlestick injuries, felt that concerns about risk had been heightened as a result.

I *think that people were just wary about going into the facility then* [after the needle sticks and infection] *they didn’t really think...I think people were just a bit scared then.* (P16).

All participants thought that their own safety should be a priority, and many found it reassuring that the mission was conducted according to this principle. Views differed, however, on how this should be operationalised. Some cited a target of 0% or 1% infection for mission success, which they understood had been politically motivated. Those who cited this figure tended to think it was meaningless or unrealistic. Those who were responsible for the safety of others (team leaders, for instance) reported operationalising their own judgements about what was risky. Some participants felt that acceptable risk was a subjective matter that was down to each individual to decide at the time.

*...my priority was keeping my people safe...I would not send my people into the facility unless there was a good reason for them to go.* (P14).

*I think that people, even in a military system, people do need to be able to opt out of a scenario where there is a 1 % risk of getting infected with a life-threatening disease...ultimately if someone doesn’t want to be there...they will find a way to go sick or they won’t do their job very well so I would rather not have someone there who did not want to be there.* (P3).

Some, however, reported having had to deal with colleagues who they thought were unduly risk averse.

*I would have described* [colleague] *as making some fairly risk-averse – surprisingly risk-averse decisions in some cases and then not in others.* (P17).

One also reported the need to rein in those who regarded undertaking invasive procedures in the red zone as a “*badge of honour*” (P18).

The management of risk was widely regarded as requiring compromises to be made in how patients were cared for and in particular how they were nursed.

*...our priority was staff health and patient health and safety but there was an element of risk aversion that made it difficult for the practitioners to feel that they were contributing fully and I can understand why that was because Ebola is a killer so you don’t want people to have too much freedom* (P9).

The participants reported that risk management presented a significant ethical challenge, and the need to balance risk to self with patient care is reflected in some of the specific ethical issues that participants discussed (Table 2).

**Table 2:** Perceived ethical challenges reported

• ‘Volunteering’ for deployment
• Number of patients being treated/empty beds
• Kinds of patients being treated – who should be regarded as a healthcare worker
• Specific types of care for the patient where balancing risks to self against providing care were especially acute. Examples included:
Use of the bowel management system
Treatment of agitated patients
Time spent in the red zone
Comforting dying patients
• End of life care and decisions
• Managing and responding to differences in risk perception, between staff, over time and in response to critical incidents
• Discharging of vulnerable patients
• Request to give convalescent blood
• Use of [the only] ventilator in the ‘red zone’
• Separation of infected healthcare professionals from their infected
• children (this treatment unit did not admit children)
• Management and disposal of bodies
• Use of cameras to monitor patients/staff
• Maintaining staff morale
• Evacuation decisions and differences in the kinds of patients who were evacuated
• Using novel equipment
• Transporting stage 3 patients over great distances
• Uncertainty how best to treat patients (because optimal treatment for EVD unclear)
• ‘Decompression’ on return
• Implementing decisions from above, the rationale for which was not clear
• Sharing of resources and facilities with NGOs
• Not being able to use/keep up skills whilst on deployment
• Persuading patients not take their own discharge

### Reactions to ‘empty beds’

The most commonly reported ethical challenge was whether the unit should have treated more patients.

Our participants reported that the treatment unit was consistently running under capacity: "*the facility was never full, it never got beyond 50% capacity and yet there were clear groups of people it wouldn’t take*". (P10). Many regarded this as a significant ethical challenge because the facilities, expertise and resources were standing idle in a sea of need.

*There we were sat in the best treatment facility in the whole of Africa, fantastic equipment and staff...products that were expiring each week and getting thrown away...sat there in a facility that I think only had about four patients in it at the time... a complete catastrophe going on all around us ...and people really struggled with that.* (P3).

*The biggest challenge was the justice component...because we had so much we could offer but we were treating hardly any patients and that didn’t sit very well with any of us because working in a place where there is lots and lots of suffering and disease and death and things and knowing that we could help but we’re not allowed to, that was awful.* (P5).

A minority disagreed with efforts to increase the number of patients admitted.

*Some of the staff got a bit anti for that because ‘Hang on a minute, why are we putting ourselves at risk you go and look after someone who’s not even on our profile list?’.* (P6).

One participant took the view that although the resources deployed seemed excessive given the number of patients treated, they may not have been disproportionate to the resources used to care for Ebola patients back in the UK. This realisation enabled the participant to take pride in what was achieved.

*...deep down as a doctor you always want...to see as many patients as possible, and help as many patients as possible, and treat as many patients as possible. I didn’t really have that choice because I was deploying on a military operation...The Royal Free is an example of – some of the physicians at the Royal Free were dealing with tiny numbers of* [Ebola] *patients, with huge numbers of healthcare workers involved in the care of one patient*. [If you take this on board] *then you don’t feel guilty but feel proud of the work that you’ve actually managed to do in your specific role.* (P21).

Most participants were able to articulate some version of both sides of the argument, but participants in favour of greater bed occupancy tended to be those who regarded themselves to be on a humanitarian mission. Views also reflected perceptions of risk outlined above. Strong feelings may have been exacerbated by the fact that the opposite problem had been anticipated, namely that the unit would be overwhelmed.

*...we kind of expected to be a bit more overwhelmed with patients, we would have perhaps to be choosing between who we had beds for and who we didn’t have beds for similar to a lot of other operations where you have your eligibility criteria* (P15).

The medical staff in particular tended to think that the integrity and spirit of the mission would have been preserved by loosening the MRoE or making greater use of the commanding officer’s discretion to accept patients outside the MRoE: "*…maybe they weren’t briefed about how restrictive the medical rules of eligibility were ... even if we were asking for patients of Ebola, we couldn’t get them in because* [of the MRoE]" (P19). Many participants were conscious that staff safety would be better preserved running at a consistent capacity since this avoided skill fade.

*...for maintaining safety of the staff it is better to have a continual level of work than it is to surge up and down with small numbers of cases...(*P2).

The feeling of being under-utilized, which was frequently associated with the MRoE, often prompted participants to comment that Operation GRITROCK was highly politicised. Many participants expressed a belief that decisions about matters of detail, including in relation to medical management, were being taken at a very high (some thought ministerial and even prime ministerial) level.

*...it was a very political deployment....in the sense that there was an awful lot of scrutiny from on high...we were having regular briefings from Cobra* [Cabinet Office briefing room A^3^]*...there was an awful lot of scrutiny from Number 10* [Downing Street], *it was the run up to the election...* (P10).

This was experienced as unprecedented and unwelcome interference particularly for clinicians used to exercising clinical judgement fairly autonomously. It is possible that this was the result of our participants’ perception that Operation GRITROCK was essentially a medical operation whereas military healthcare personnel normally deploy to support combat missions, meaning that clinicians are more insulated from higher-level political preoccupations. Whatever the cause, the effect was that some participants in all groups were left with the impression that those on the ground were unable to take responsibility for, or justify, the decisions being made, leaving them to implement decisions that, as far as they were concerned, did not make sense in the context of their understanding of the mission. This was also a source of perceived ethical tension.

*I mean we are in the armed forces, it is not a democracy you know. If someone just is honest and says: ‘no this decision has been made end of, just put up with it’, then although we might not be happy with it, we will put up with it because we know that’s the organisation. But to obfuscate because someone had made a decision that people may be ethically unhappy with but they are not willing to say, ‘yes, I have made that decision and I am going to stand by it’, is something that was very frustrating, we didn’t know at what stage those decisions were really being made or enforced.* (P4).

### Points of reference for participants’ values

A shared understanding of what was meant by an ethical challenge was established either during the interview or immediately before it commenced. We took as our working definition that adopted by Schwartz et al.: ‘situations where either the HCPs [health care professionals] knew what they felt was the right thing to do but were somehow prevented from enacting it, or where “doing the right thing” also caused harm’ [4].

Their values and norms manifested themselves in either how participants perceived ethical challenges to have arisen – where they felt unable to act in accordance with their values – or in how they perceived and addressed the specific ethical challenges (see Table 2 above) they faced. Only a few participants used terms (the technical language of ethics) that spoke directly to specific ethical principles or values.

Our participants’ understanding of what ‘the right thing’ was tended to be informed by values and norms derived from a combination of:

i. Previous deployment experience

For most (but not all) participants this was combat experience. Previous deployment experience was influential in shaping expectations and norms with regard to the application of MRoE and risk perception (see previous section on risk) but was also referred to in relation to specific issues such as the dignified handling of human remains.

*I have worked in resource limited settings before and certainly in Afghanistan… we had at least two patients who would have been dialysed if they’d been in the UK and who died because we did not have any dialysis available. So I am used … to dealing with that and the concept that you can’t give people care that you don’t have available.* (P1).

*Previously on tours in Afghanistan that I’d done, the bodies were handled with a lot of dignity afterwards*. (P22).

The bodies of those who die from EVD are highly contagious. This meant that the usual rituals around death (both for staff and in terms of the local culture) could not be observed. The loss of these rituals added to the discomfort of staff.

Deviations from previous experience created uncertainties that, where unresolved, generated what our participants perceived as ethical challenges.

*I am not naive enough to think that at an operational level you truly understanding the wider picture but if you are...on the front line implementing the decisions that are made higher up...I did enforce them* [MRoE] *the year before when I was in ... Afghanistan...and that was a very difficult decision to make...but I never had so many struggles with what I felt was unethical.* (P2).

ii. NHS/UK experience

Similarities with familiar practices were a source of reassurance and dissimilarities prompted reflection if not discomfort.

*Because deep down inside you know that you should be, you know, there’s this patient does he need a bowel management system? Well actually no he wouldn’t do if he was back in the UK because you wouldn’t do it because it benefits us ‘cause we have to go in and change him all the time.* (P13).

*There was an understanding that we’d prioritise the military side first but...I see a child of say seven years old from the Save the Children side, in my mind, I prioritise, just like the NHS, I prioritise the child*. (P19).

Another example was the rationale P21 recalled above that “*the Royal Free were dealing with tiny numbers of [Ebola] patients, with huge numbers of healthcare workers involved in the care of one patient*.” This perceived similarity with the situation in the treatment unit enabled that participant to feel pride in what was achieved.

iii. Professional values

Professional values were a clear source of guidance as one might expect but also create the ‘dual obligation’ problem. This participant, for instance, thought that adherence to professional values was more important than following orders, and indeed may be a measure of the legality of orders.

*You’ve kind of got two sets of rules for want of a better word that you kind of have to abide by...I have my Code of Conduct, the NMC* [Nursing and Midwifery Council] *Code of Conduct. I would like to think that if I was asked to do anything militarily that came into conflict with that NMC code I would be able to stand up and say ‘No’ and that you could then put that down to erm an unlawful order that’s been given you.* (P11).

iv. Military values

Unsurprisingly, our data suggest that our participants were highly conscious of being military personnel. References to values, including military values, were often not explicitly expressed or identified but can be inferred from the sentiments being expressed by the participants. A summary of UK military values can be found in Table 3.

**Table 3:** UK military values

The three services that comprise the UK military, The Royal Air Force, the Royal Navy and the Army, articulate their own values and ethos. In summary, they are as follows:
Royal Air Force’s ‘Core values and standards’ [14] are: **Respect –** Mutual and Self Respect **Integrity –** Moral Courage – Honesty – Responsibility – Justice **Service –** Physical Courage – Loyalty – Commitment – Teamwork **Excellence –** Personal Excellence – Discipline – Pride
Royal Navy’s ‘Core Values’ [15] are: commitment, courage, discipline, respect for others, integrity, loyalty
The Army’s ‘Values and Standards’ [16] are: loyalty, integrity, courage, discipline, respect for others and selfless commitment
The Royal Marines (part of the Royal Navy) has its own distinctive ethos and values. [17] The Royal Marines ethos is made up of the individual ‘Commando Spirit’ (courage, determination, unselfishness, and cheerfulness in the face of adversity) and the collective ‘Group Values’ of courage, unity, determination, adaptability, unselfishness, humility, cheerfulness, professional standards, fortitude and commando humour.

Some participants expressed trust in, or at least acceptance of, the chain of command, although for others Operation GRITROCK undermined this trust. A sense of obligation within the chain of command resulted in paternalistic benevolence to those under one’s command, such as bolstering troop morale and serving their interests (and this may at least partly account for the trust in the chain of command).

*As their boss, it’s my job to look after them and keep them safe...in my previous roles, I’ve always taken my responsibility to looking after my soldiers very seriously...I will always refer to my personnel as soldiers; I think of myself as a soldier...It does make me fiercely protective of my soldiers...where the risk and the danger was so real...it directly affected us it just makes my fiercely protective of my people.* (P14).

Participants generally demonstrated a willing, but not blind, adherence to ‘the mission’. As we have seen in relation to motivation and risk perception the nature of the mission was itself a yardstick against which to measure the right response to situations. Some participants, like P17** above (‘*we can take that risk and so that the UK can remain safe’*), clearly expressed the view that being in the military meant being in service to, and taking risks on behalf of, the nation. As we have seen already, again in relation to risk, (for instance, P13*** above: “*I felt confident that as long as I stuck to my ‘drills and skills’ and we looked after each other, we’d be fine”*) there was also a strong identification with a team or unit. In this respect, the ‘self’ was regarded as part of, important to, and protected by the collective or team.

*you can’t do rushing in... you can’t do anything more than you can do. You take it slowly because...accidents to our own staff then compromise even more people because then you’ve got a situation where you’ve gotta send another team in to get them out.* (P12).

Although many regarded the mission as humanitarian, participants did not refer specifically to the humanitarian codes of conduct such as those advocated by Sphere [18] or the International Committee of the Red Cross [19, 20]; though the United Nations Office for the Coordination of Humanitarian Affairs (UNOCHA) humanitarian principles [21] are listed in relevant doctrine (the 3rd edition, most current edition of which was published December 2016 but the principles are also listed in the 2nd edition published 2012) [22]. These do, however, have much in common with e.g. professional values. One participant seemed, however, to have some familiarity with the humanitarian principles and had come to question whether the military should have a role in humanitarian missions as a result.

*When we do a purely military operation, you know, I believe our role is to look after our service men, as military doctors to look after them...someone said to me recently ‘well couldn’t we shape the military to be more of a humanitarian response?’ and after GRITROCK I think we shouldn’t; I don’t think we have much of a role to play in humanitarian response because of the lack of adhering to kind of basic humanitarian principles – independence, humanity* etc.*..* (P2).

## Discussion

Perceptions about volunteering were prevalent in both reservists and regulars, even though it is only reservists who have any, albeit limited, control over the timing of their mobilisation [23]. It is possible that the sense of being a volunteer arose because traditional high readiness units are trauma-centred and different skills are needed to tackle an infectious disease outbreak. Accordingly, many of those who initially deployed with the high readiness unit were infectious diseases/infection control specialists or others perceived to have skills in relation to general nursing, palliation or achieving difficult venous access, and who were not ‘scheduled’ to deploy or part of the high readiness unit at the time. Many of our participants actively sought or welcomed deployment but it is not obvious how much weight would have been given to reluctance had it been expressed and if no replacement was available. Nonetheless, our data suggests that the moral motivations expressed by our participants, which align more with NGO workers than has sometimes been suggested, [24] should be acknowledged. Given that some humanitarian disasters are a direct result of armed conflict, the strong pull felt by some of our participants to undertake humanitarian work may seem at odds with the decision to join the military. This may be the result of historical recruitment campaigns that appeared to emphasise humanitarian action, [25] even though – in the experience of our participants, at least – it has not been a core activity in recent years.

This sense of volunteering is further testament to the sense of duty felt by some healthcare workers to provide care despite the personal risks of doing so. Trust in military training (‘drills and skills’), colleagues and military infrastructures and the PPE provided was a significant factor in addressing concerns about risk. There may be lessons here for civilian health services preparing for domestic emergencies, including pandemics and bioterrorism.

The discomfort reported in relation to the application of the MRoE was not surprising. It is a typical manifestation of the dual obligation problem: healthcare professional or soldier first? Across the data set, our participants provided no consistent response to this question. Some clearly identified more with their military obligations and others with their professional obligations. This may in part be explained by the fact that some participants had moved from conventional military combat roles into more healthcare focussed roles, for which they undertook conventional professional training and gained professional qualifications. Others gained their professional qualifications before joining the military, and others qualified alongside maintaining a reservist role. Some participants seemed reconciled to the tension in their different roles. This is consistent with Gordon’s findings that military doctors forge an identity by finding ways of bridging the two alternative sets of obligations [26].

Dual obligations form only part of the problem. The UK medical military exists to support the military population at risk, but must also operate according to United Nations (UN) agreements. Of particular relevance in the case of Operation GRITROCK are the UNOCHA guidelines [27] and the ‘Oslo guidelines’, [28] which require humanitarian activities to be sensitive to an indigenous government’s own efforts to maintain infrastructures. This principle applies equally to civilian responders. Access to UK medical military facilities are always governed by MRoE, which ensure facilities operate without undermining the ‘military mission’, international agreements or codes of professional conduct. This is a difficult balance, compounded for military healthcare personnel by their professional imperative to offer treatment to those in need. It is one that invariably produces controversy, [29] and Operation GRITROCK was no exception [30, 31]. The ethical disquiet for military healthcare personnel is particularly acute when they have spare capacity and resources. The MRoE may deny help to those who need it and create ‘dual standards of care’ (military facilities, for instance, may be superior - in terms of specialised and experienced staff and resources - to local facilities) [29, 32]. Military medical personnel, as indicated in our data, are accustomed to circumstances where capacity in combat missions needs to be kept in reserve for a potential influx of wounded personnel. In this deployment, the corollary population were eligible patients, particularly *international* healthcare and other staff, thereby making good on the undertaking to provide a safety net for expatriates helping the local population. Given that the participants understood the mission and did not appear to reject the whole notion of MRoE, it is worth reflecting a little further on the discomfort expressed in this context.

There was a great deal of UK media coverage of the scale of the Ebola threat, and the human suffering being left in its wake. Our participants, like people the world over, had been following the unfolding crisis. The deployment of UK troops also received considerable attention. The proportion of local healthcare workers affected by EVD had, however, peaked over the summer of 2014 [33]. The treatment unit admitted 125 patients from its opening in November 2014 to June 2015: of these 43 were confirmed to have EVD and only one confirmed EVD patient was treated in the unit after 1st April 2015 [34]. A highly motivated and primed workforce believing itself to be on a humanitarian mission therefore found itself under-utilised, but conscious that it occupied an extremely well-resourced facility running alongside a local system that was still over-stretched. This greatly exacerbated the usual tensions over MRoE. Some participants felt that once the peak had passed, the superior facilities they were able to offer should have been made available to anyone infected with EVD in Sierra Leone so as to improve their prospects of survival and comfort. Such an extension to the mission at this point may, however, have breached the Oslo guidelines (for instance, by potentially undermining local services). This tension was not a uniquely military/medical one. An NGO-run, similarly situated unit would face the same dilemma at this point in an epidemic. Indeed, NGO healthcare workers’ practice may also be additionally constrained, not by military imperatives but by donor expectations and tightly defined/negotiated missions [35, 36]. Arguably, constraints on practice such as these are further common ethical ground between NGO and military responders.

Although some of our participants reported feeling very conflicted as a result of not being able to do more when they had the capacity and resources to do so, it is worth noting that the international decision to deploy troops to West Africa may well have bolstered the confidence and resolve of civilian responders, [37] which was the rationale behind establishing the treatment unit [3] and the MRoE under which it accepted patients. Nonetheless, the feelings of distress, anger and impotence created by the empty beds had clearly remained with our participants several months later.

In terms of military training and preparation, lessons can be learned from the difference in perception of the ethical challenges experienced that resulted from the differences in perception about the essential nature of the mission (humanitarian/military), by exploring and critiquing the likely sources of ethical values (military, professional and based on deployed and non-deployed experiences) and the potential for ethical distress where the reasons for decisions are not fully explained/shared.

Discussing likely scenarios and potential resolutions and their relative advantages and disadvantages is an established way of preparing healthcare workers to meet the ethical challenges they may face in their workplace. The experiences of our participants were a rich source of data for such scenarios, which could be generalised beyond the military context and also generalised to other serious infectious disease outbreaks. Such scenarios could be incorporated into the training and preparation of future healthcare professionals and other health-related staff (military and civilian) for the ethical challenges that they may face on humanitarian deployment. A series of fictionalised case studies were therefore created, based on composite experiences of our participants. These scenarios (along with some notes to aid e.g. group discussion or self-directed learning) can be found on the project website: https://www2.warwick.ac.uk/fac/med/research/hscience/sssh/newethics/bioethics/milmed/ebola/caseteaching/.

### Limitations

It is important to recognise that these findings are based on a qualitative study that explored the subjective views and perceptions of those who participated. It was not an investigation or inquiry aimed at determining specific facts about the military deployment. It offers an insight into how those who participated reportedly felt about their experiences. The nature and purpose of the study was advertised in the information sheets and there may therefore be some degree of volunteer bias in the responses. We do, however, feel fairly confident that saturation in this group was achieved. The semi-structured nature of the interview guide may have had a ‘framing’ effect.

## Conclusion

This study offers unique insights into the military healthcare workers’ experiences of humanitarian deployment, and these experiences were gained in the unprecedented context of the first major Ebola outbreak. Those interviewed expected the treatment unit to be overwhelmed and the empty beds presented a significant and pervasive ethical challenge for them, particularly for those professing humanitarian motivations. The different perceptions of the mission (humanitarian/military) gave rise to different perceptions of the ethical challenges being faced. Participants’ judgements were also influenced by values from four principal sources, including specific military values that might not be shared by civilian humanitarians. Many participants, however, expressed motivations that gave them common purpose with NGO volunteers.

We are currently exploring with the military (and civilian organisations) how to best to ensure that the lessons identified in this exercise are incorporated into policy and practice going forward.

## Abbreviations

Cobra: Cabinet Office briefing room A
DFID: Department for International Development
DMS: Defence Medical Services
ETC: Ebola treatment centre
ETU: Ebola treatment unit
EVD: Ebola virus disease
HCPs: Health care professionals
HD: Heather Draper
IEDs: Improvised explosive devices
MODREC: Ministry of Defence Research Ethics Committee
MRoE: Medical Rules of Eligibility
NGO: Non-governmental organisations
NHS: National Health Service
NMC: Nursing and Midwifery Council
PPE: Personal protective equipment
RCDM: Royal Centre for Defence Medicine (Academia and Research)
SJ: Simon Jenkins
UK: United Kingdom
UN: United Nations
UNOCHA: United Nations Office for the Coordination of Humanitarian Affairs
WHO: World Health Organisation

## Footnotes

1 Medical Rules of Eligibility (MoRE) are the rules governing who will be given access to UK military facilities, taking current capacity and existing and upcoming combat operations into account. UK and allied troops generally have access to all facilities, as may non-local nationals from allied nations. Local nationals may be treated under some circumstances and according to capacity. For more information on UK MRoE see *Ministry of Defence (Development, Concepts and Doctrine Centre) Joint Doctrine Note 3/14 The Military Medical Contribution to Security and Stabilisation. Crown Copyright June 2014.* *https://www.gov.uk/government/uploads/system/uploads/attachment_data/file/324637/20140616-JDN_3_14_Med_contr_DCDC.pdf* [Accessed 26th June 2017]

2 Simulation training prior to deployment included a replica of the treatment unit and simulated care, treatment and investigations of ‘patients’ complete with simulated bodily fluids. The bodily fluids included titrated amounts of ultraviolet dye. The dye represented the virus found in the bodily fluids of infected patients and the titration simulated different levels of infectiousness. Prior to and after removal of their PPE, trainees were scanned using a hand-held ultraviolet lamp. This revealed any traces of the ultraviolet dye signifying areas of potential contamination, which were formally mapped before and after doffing PPE. Thus the extent to which the PPE had contained the simulated contamination was immediately apparent, as well as providing potential quality assurance and the ability to improve on doffing and decontamination techniques where any breach was noted.

3 Cobra stands for ‘Cabinet Office briefing room A’. It part of the UK governments’ civil contingency planning and coordinates the emergency response across government departments. The composition of the group varies according to the emergency. Cobra is often but not always chaired by the Prime Minister.
